# Passive Surveillance as a Key Tool for African Swine Fever Eradication in Wild Boar: A Protocol to Find Carcasses Tested and Validated in the Mediterranean Island of Sardinia

**DOI:** 10.3390/v16010136

**Published:** 2024-01-18

**Authors:** Elisabetta Coradduzza, Federica Loi, Francesca Porcu, Daniela Mandas, Fabio Secci, Marco Efisio Pisanu, Cinzia Pasini, Carlo Zuddas, Marcella Cherchi, Daniele Denurra, Ennio Bandino, Antonio Pintore, Vittorio Guberti, Stefano Cappai

**Affiliations:** 1 Istituto Zooprofilattico Sperimentale della Sardegna, 07100 Sassari, Italy; elisabetta.coradduzza@izs-sardegna.it (E.C.); marcella.cherchi@izs-sardegna.it (M.C.); danieledenur@virgilio.it (D.D.); ennio.bandino@izs-sardegna.it (E.B.); antonio.pintore@izs-sardegna.it (A.P.); 2 Osservatorio Epidemiologico Veterinario Regionale, Istituto Zooprofilattico Sperimentale della Sardegna, 09125 Cagliari, Italy; francescaporcu12@gmail.com (F.P.); daniela.mandas@izs-sardegna.it (D.M.); cinzia.pasini@izs-sardegna.it (C.P.); carlo.zuddas@izs-sardegna.it (C.Z.); stefano.cappai@izs-sardegna.it (S.C.); 3 Local Sanitary Agency of Sulcis Iglesiente, 09013 Carbonia, Italy; fabiosecci@aslsulcis.it; 4 Associazione CPT—Caccia Pesca e Tradizioni Sardegna, 09094 Marrubiu, Italy; marcoefisiopisanu@gmail.com; 5 Institute for Environmental Protection and Research (ISPRA), 00144 Roma, Italy; vittorio.guberti@isprambiente.it

**Keywords:** African swine fever, passive surveillance, carcasses, eradication, mobile app, freedom from animal disease, wild boar

## Abstract

African swine fever (ASF) is one of the most important and serious contagious hemorrhagic viral diseases affecting domestic pigs and wild boar and is associated with high mortality rates while also having an extensive sanitary and socioeconomic impact on the international trade of animal and swine products. The early detection of the disease is often hampered by inadequate surveillance. Among the surveillance strategies used, passive surveillance of wild boars is considered the most effective method for controlling the African swine fever virus (ASFV). Otherwise, the design of a sufficiently sensitive ASF surveillance system requires a solid understanding of the epidemiology related to the local eco-social context, especially in the absence of virus detection. Even if the number of carcasses needed to demonstrate ASF eradication has been established, the scientific context lacks detail compared to protocols applied in the active search for wild boar carcasses. The aim of this study was to describe the protocol applied in the active search for carcasses, providing detailed information on the number of people and dogs as well as the amount of time and space used within the Mediterranean area. Using a specific tool developed to record, trace, and share field data (the *GAIA observer* app), a total of 33 active searches for wild boar carcasses were organized during 2021–2023. Most of these searches were planned to find carcasses that had previously been reported by hunters. A total of 24 carcasses were found, with only 2 carcasses not previously reported. The final protocol applied involved four people, with an average speed of 1.5 km/h. When a carcass had been previously reported, about 2 km of distance had to be covered in about 1.5 h to find the carcass, and even less time was spent when a dog (untrained) was present. In conclusion, it can be stated that, when searching for carcasses, solid collaboration with local hunters or other forest visitors is necessary to ensure carcasses are reported. The process involves small groups of experts actively searching for carcasses, possibly with the use of hunting dogs without special training. The data presented could be of valid support for those countries characterized by Mediterranean vegetation that are faced with the need to plan active carcass searches.

## 1. Introduction

Caused by a large double-stranded enveloped DNA virus of the *Asfarviridae* family, African swine fever (ASF) is a fatal infectious disease that affects wild and domesticated suids [1]. Most ASFV infections are associated with acute hemorrhagic fever, with a case fatality rate approaching 100% in domestic pigs and wild boar [2]. The epidemiological characteristics of ASF and the current spread of the disease pose a global threat that can be described as the most serious animal health emergency the world has ever faced [3]. The absence of a vaccine, the rapid spread between continents, and the disease’s ability to affect both domestic and feral pigs make its control and eradication even more difficult [4,5]. The carcasses of infected wild boar, which gradually degrade in forests, seem to play a significant role in the spread of the African swine fever virus (ASFV) [5,6,7,8]. Their consumption by other susceptible individuals (i.e., wild boar and feral pigs) has already been demonstrated, and consequently, the virus’s correlations with disease spread [9,10]. Thus, rapidly finding and removing carcasses is a crucial measure for effective ASF control [11]. 

ASF has been endemic in Africa since 1921 and is reported in 32 African countries [12]. As a result of the first spread of ASFV genotype I in Europe in 1957–1960, the Italian island of Sardinia was classified as the only ASF-endemic area on the European continent for more than 40 years [13,14]. Since 2007, ASFV genotype II has spread across the European continent, starting in Georgia (2007) and recording significant outbreaks in neighboring countries, including Armenia and the Russian Federation (2007), Ukraine (2012), Belarus (2013), the Baltic states, and Poland (2014). In 2017 and 2018, ASFV was detected in either wild boar or domestic pigs in the Czech Republic, Romania, Hungary, Moldavia, and Belgium [15,16,17,18,19,20,21]. More recently, ASFV genotype II outbreaks were notified in Italy, where long-distance jumping transmitted the virus from infected northern regions to previously ASF-free southern areas [22,23].

To date, ASF has spread to over 50 countries across Africa, Asia, Europe, the Pacific, and the Caribbean since 2007 [24]. Since January 2021, 10 countries have reported a preliminary occurrence of ASF as a first occurrence in their country, while 12 countries have reported its spread to new zones. This highlights the continuous spread of the disease into new countries and new zones in countries already affected.

Disease surveillance in the EU is addressed by early detection in ASFV-free areas and the implementation of specific control measures in areas defined as endemic [25]. Among the implemented control measures, passive surveillance (i.e., sampling and testing the sick or dead animals) was identified as the target method for the early detection of the disease by the European Food Safety Authority (EFSA) [17]. Indeed, as previously reported, most of the primary cases of wild boar were found via passive surveillance [17,26,27,28]. Otherwise, in most European countries, the surveillance of wild populations is mainly based on active surveillance (i.e., testing all hunted wild boar for viruses and antibodies) during wild boar hunting [17]. Passive surveillance should be preferred, even in the final stage of the disease eradication, meaning the point at which the virus is no longer detected but seropositive animals are still present [29], or in endemic areas under the conditions of disease endemicity and low prevalence, as provided in EU Directive 7113/2015 [30]. Indeed, seroprevalence in adult wild boar would be a poor indicator for demonstrating the absence of virus circulation [29]. Thus, each country facing the last phases of ASF eradication must provide evidence of virus absence by applying a specific exit strategy [29]. The strategy should be mainly based on passive surveillance rather than active surveillance, given that the inclusion of active surveillance in the exit strategy only marginally improves its performance [29,31,32]. Specifically, target passive surveillance via the active search for carcasses in a specific timeframe and in at-risk areas is strongly suggested in the scientific opinion of the EFSA to provide timely evidence of virus eradication [29,32]. However, a strategy based on passive surveillance will only be effective if farmers’ and hunters’ disease awareness is sufficiently high, whereby a suspicion based on observed mortality is followed by timely reporting to the competent authorities [33]. Instead, passive surveillance is often limited to poorly planned sampling, resulting from the activities of inadequately trained volunteers and citizens or from samples taken following road accidents [29,30,31,32,33,34]. In their scientific opinion, the EFSA estimates the need to report, sample, and test a number of carcasses each year (wild boar dead from non-hunting causes but not those killed in road traffic accidents) equal to about 1–2% of the estimated wild boar population [29]. Thus, for example, in Italy, at least 5000 wild boars per year should be tested for a population of at least 500,000 animals in the pre-reproductive period [35,36]. Even if the EFSA exit strategy provides guidelines on the number of carcasses that must be found and tested as ASFV-negative to declare the disease eradicated, the opinion did not provide any indication of the practical actions to carry out when passive surveillance must be put into place [29]. 

To date, Sardinia is the only ASF-infected country that has elaborated and completed its specific exit strategy based on the active search for carcasses and following the EFSA guidelines [37]. Indeed, upstream from the previously mentioned ASF pandemic context, Sardinia’s wild boar population has recently been declared free from ASFV genotype I [38]. After 37 years of repeated endemic picks, the eradication program against ASF put in place in 2015 was successful [39,40,41]. Even if the genotype I strain circulating in Sardinia has always been associated with high virulence, the virus was recently isolated from apparently healthy pigs, suggesting the presence of a less virulent ASFV [29]. A drastic decrease in disease prevalence started in 2017 and became even more evident in 2020, occurring mainly due to the culling actions against free-ranging pigs [42]. The last virus detections date back to 2018 and 2019 and were found in domestic pigs and wild boar, respectively [43]. The last two wild boars detected as ASFV positive were found dead, and their identification was followed by a sequel of seropositive cases mainly detected in adult and subadult individuals [29,37]. In 2020, the Sardinian Region put in place a strong surveillance plan based on the active search for wild boar carcasses with the aim of providing evidence of virus extinction. Three years later, after the completion of the ASF exit strategy via passive surveillance, the island received the coveted seal of ASF-free status in the wild boar population [38]. 

The aim of this paper is to describe the strategy applied for passive surveillance implemented in Sardinia by referring to (1) the identification of the most suitable areas to find wild boar carcasses; (2) the number of carcasses to be found to provide evidence of an absence of disease; (3) the protocol applied in the field (i.e., the number of people and dogs, as well as the amount of time and space); and (4) the fundamental role of citizen science and participatory methods, with a focus on the role of the hunters in finding wild boar carcasses. The on-field data described here are among the few examples of the active search for carcasses in Mediterranean areas and could be an essential starting point for those countries characterized by Mediterranean vegetation that are faced with the need to plan an active search for carcasses.

## 2. Materials and Methods

### 2.1. Study Area

**Figure 1:**
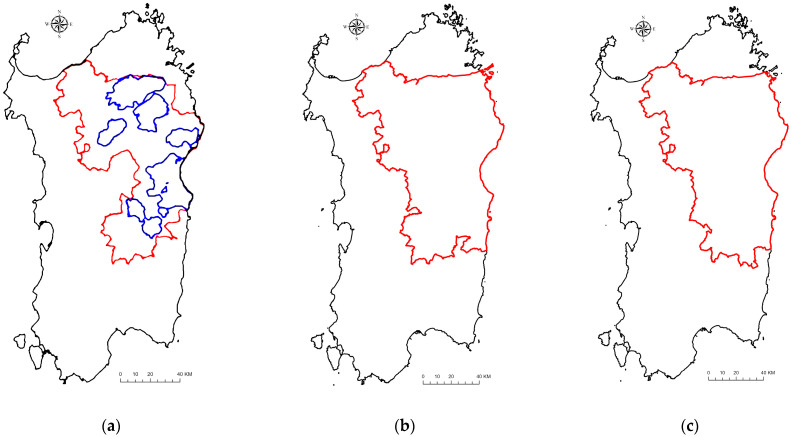

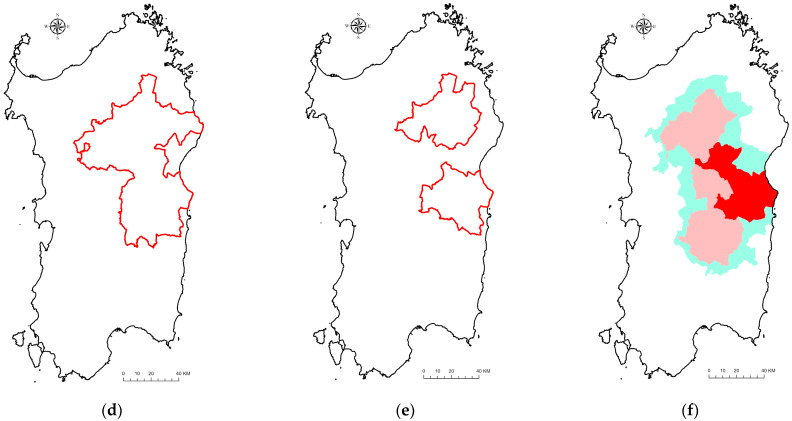
Excursus of the Sardinian-wild-boar-infected areas from 2012 to 2022 and restriction zones for 2023. (**a**) Infected area instituted in 2012 (blue limits) and subsequent enlarged infected area established in 2015 (red limits); (**b**) infected area for 2017; (**c**) infected area for 2018; (**d**) infected areas for 2021; (**e**) two separated infected areas established in 2022; and (**f**) ASF risk areas, including as restricted zones in Part I (green area), Part II (pink area), and Part III (red area) of Annex I.

Although the island has been affected by ASF for 43 years, infection in the wild boar population has always been limited to an area located in the northeast [40,44]. The first wild boar infection area, defined in 2012, was composed of eight different macro-infected areas (the blue areas in Figure 1a), spanning an area with a total size of 2521 km^2^. The area was then increased to 8021 km^2^ in 2015 (red area in Figure 1a) and subsequently updated every year based on the epidemiological disease trend. In 2017, a larger infected area was established, which comprised 124 municipalities and spanned a total of 9327 km^2^ (Figure 1b). After 2017, given the decrease in ASF prevalence (both virological and serological), the infected area shrank to 9299 km^2^ in 2018 (Figure 1c) and to 5322 km^2^ in 2021 (Figure 1d). In 2022, the infected area was split into two different areas, the north and south areas, based on the clusters of seropositive animals detected during the previous hunting season. These areas were 1760 and 1602 km^2^, respectively (Figure 1e). About 6000 pig farms were included in the infected areas and subjected to strong control measures [40,45]. In May 2023, the European Commission modified the Sardinian status and established three different geographic areas subject to special disease control measures. These included ASF risk areas in Parts I, II, and III, represented in Figure 1f. For the first time in 43 years, the Sardinian pig and pig production trade were allowed, following EU directives. Finally, in October 2023, the European Commission declared the Sardinian wild boar population free from ASFV and the eradication of ASFV from the wild boar [38].

### 2.2. Sardinian Wild Boar Population

Sardinia is an Italian island with an area of 24,100 km^2^ and is characterized by the typical Mediterranean climate and vegetation. The estimated Sardinian wild boar population is about 100,000 animals, with a mean density of 4.5 wild boar/km^2^ [46]. Wild boar hunting is allowed only under derogation between November and January, with an estimated population turnover of about 45% [37]. All the wild boar hunted inside the infected area must be tested for ASFV by a real-time polymerase chain reaction (RT-PCR), and serological tests are carried out using an enzyme-linked immunosorbent assay (ELISA) as a screening test, with confirmation performed via immunoblotting [47]. The primary role of wild boar in the Sardinian epidemiological cycle of ASF involving three suid populations (i.e., domestic pigs, wild boar, and illegal free-ranging pigs) has never been recognized [40,48]. The wild boar population has always had a secondary role in the maintenance of disease endemicity in the absence of a continuous source of virus [49]. Notwithstanding, the contribution of WBs to ASFV maintenance is explained by contact with the FRP population via live or dead animals (carcasses). In April 2019, the last wild boar PCR positives were identified and were followed by a sequence of seropositive cases detected mainly in adult and subadult individuals. Until 2019, only active surveillance was in place, and prevalence data referred only to wild boar hunted or killed in road traffic accidents [29,37,43]. In 2020, after about one year of the virus’ absence, passive surveillance was implemented all over the island, applying a context-specific EFSA exit strategy [37].

### 2.3. Wild Boar Passive Surveillance

**Figure 2:**
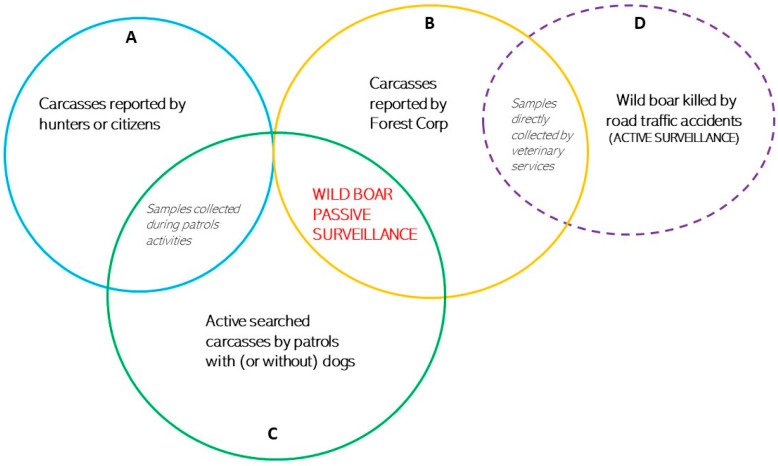
Graph representing the passive surveillance components: (**A**) carcasses reported by hunters or citizens, (**B**) carcasses reported by Forest Corp during patrol activities, (**C**) carcasses found by organized active search with or without dogs, and (**D**) wild boar killed by road traffic considered active surveillance.

As illustrated in Figure 2, passive surveillance includes different means of carcass detection. For the purposes of this work, the wild boar carcasses that fell under passive surveillance could be clustered as follows:

1. Carcass type A: reported by hunters or other forest visitors (i.e., farmers, mushroom gatherers, campers, or common citizens);
2. Carcass type B: reported by Forest Corp during patrol activities;
3. Carcass type C: found in the forest during active search with or without dogs.

Otherwise, the discovery of wild boar after being killed by road traffic (carcass type D) is considered to be a form of active surveillance, as suggested by the scientific opinion EFSA [30].

Carcasses reported by Forest Corp during patrol activities, as well as carcasses of wild boar killed in road traffic accidents, are immediately sampled by veterinary services. If a carcass is identified by hunters or citizens during hunting activities or forest visits, it is reported to the Epidemiological Observatory of IZS-Sardegna, which organizes an active search for these carcasses. During these searches, the carcasses are collected and sampled. Furthermore, an active search is organized in the field to find and sample wild boar carcasses that have not been previously reported.

### 2.4. How Many Carcasses and Where? 

As previously defined by Cappai et al., 2022 [37], the protocol put in place for passive surveillance was based on the number of carcasses to be collected and tested in Sardinia to provide evidence of ASFV absence. Using the *WBC counter tools* developed in [37] (available at: https://r-ubesp.dctv.unipd.it/shiny/WBC-counter/, last access: 3 January 2024), a wild boar density approach was applied to establish the number of carcasses that should have been detected and tested as ASFV-negative to declare the area free from ASF during the screening and confirmatory phases. The strategy was elaborated for each one of the three Sardinian wild boar hunting management units (HMUs), included (totally or partially) in the infected area: Goceano–Gallura, Nuoro–Baronia, and Gennargentu–Ogliastra. Setting parameters are reported in Table 1. 

Outside the infected area, a representative sampling of the wild boar population was estimated, given that the EFSA exit strategy cannot be applied because of its assumptions [29]. Considering an overall population equal to 68,000 wild boars in Sardinia, an estimated yearly carcass population equal to 3450 wild boars naturally dies outside the infected area. To obtain a representative sampling by passive surveillance, 1% of these carcasses (35 dead wild boars) should be found and tested for ASFV every year.

In 2021–2023, active searches for carcasses (carcass types A or C) were planned in specific areas. As this was carried out in several countries, a specific distribution model was applied to investigate whether the fine-scale distribution of ASF-infected animals can be predicted or support wild boar carcass searches [50,51,52]. The results obtained through the models led to the creation of a graphic tool providing specific indications about areas where the active search for carcasses was a priority [37,43]. 

### 2.5. The GAIA observer app 

**Figure 3:**
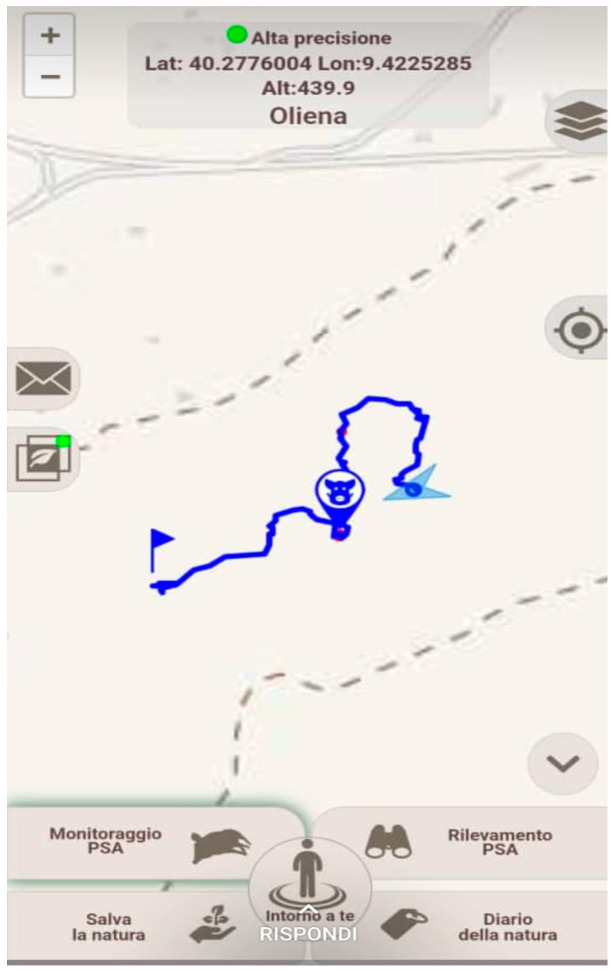
Gaia observer mobile app screen.

A specific mobile application (app) called “*GAIA observer*” was developed by Xvalue SRL^®^ to be compatible with Android, iOS, and Windows. Combining a platform with a mobile app makes it possible to collect several pieces of georeferenced information from the environment. The *Gaia observer* app only requires a GPS position to function and allows researchers to download maps of all Italian regions free of charge (Figure 3). 

The app was created for the purpose of data collection from ASF passive surveillance project activities, particularly for the reporting of carcass types A or C, and it was developed in Italian to facilitate users in reporting findings and sightings in real time. 

The license was provided to IZS-Sardinia. The online app makes it possible to download data previously uploaded by users and to monitor the real-time data forwarded by users in the field.

The platform reserved for ASF passive surveillance includes the following:

i. *Report database*: this includes the carcass code, the date the carcass was found, the name of the operator who made the finding, municipality, province, region, latitude, longitude, altimetry, track code (unique code), and photos of the carcass;
ii. *Real-time monitor report*: this provides a real-time view of carcass findings with the date the carcass was found, the carcass code, and the carcass coordinates;
iii. *Route database*: this contains the details of each field patrol: the name of the operator, region, province, common, date, start time, end time, duration, and length (m) of the route;
iv. *Real-time route monitoring*: this shows the geographical map of Sardinia, allowing operators in the field to be monitored in real time.

**Figure 4:**
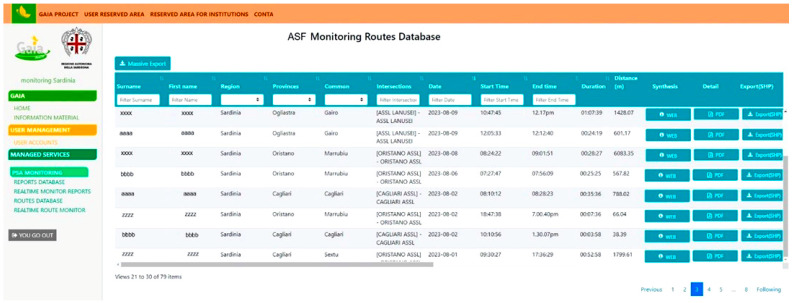
The online interface of the *GAIA observer* app, with a focus on the route database. The data collected referred to the user’s name and surname (encrypted to guarantee the people’s privacy), location information (region, province, common, and ASL intersection in the territory), time spent, and distance traveled.

An interface of the app in English is shown in Figure 4. 

### 2.6. The Active Carcass Search Protocol 

The following protocol was applied to find carcass types A (reported carcasses) or type C (carcasses not previously reported and found by active search).

Each team was composed of a veterinarian from IZS-Sardegna, field experts from local hunting companies, and veterinarians from the local veterinary service (ASL). Trained dogs and their owners had the option to participate in the search.

Each on-field activity was planned and organized based on a specific calendar that was shared and approved by each team category coordinator. Furthermore, after the identification of the target areas, a report was sent to each coordinator. For carcass type A (Figure 2, bundle A), the area was selected according to previous notifications from hunters or forest visitors. For carcass type C (Figure 2, bundle C), the areas were selected according to the distribution model. The report on the characteristics of the area (i.e., shape files, coordinates, vegetation, density of wild boar, presence of farms or private properties). 

Before beginning the search activities, each person was required to consent to the use of their name, surname, affiliation, and contact information (i.e., telephone number). This was essential in case there was a need to contact the users in the case of data inconsistency, doubts, or errors in the data collected. Subsequently, the user was required to download the *GAIA observer* app on their personal mobile device for real-time data collection (routes, times, patrolled areas, and carcasses found).

**Figure 5:**
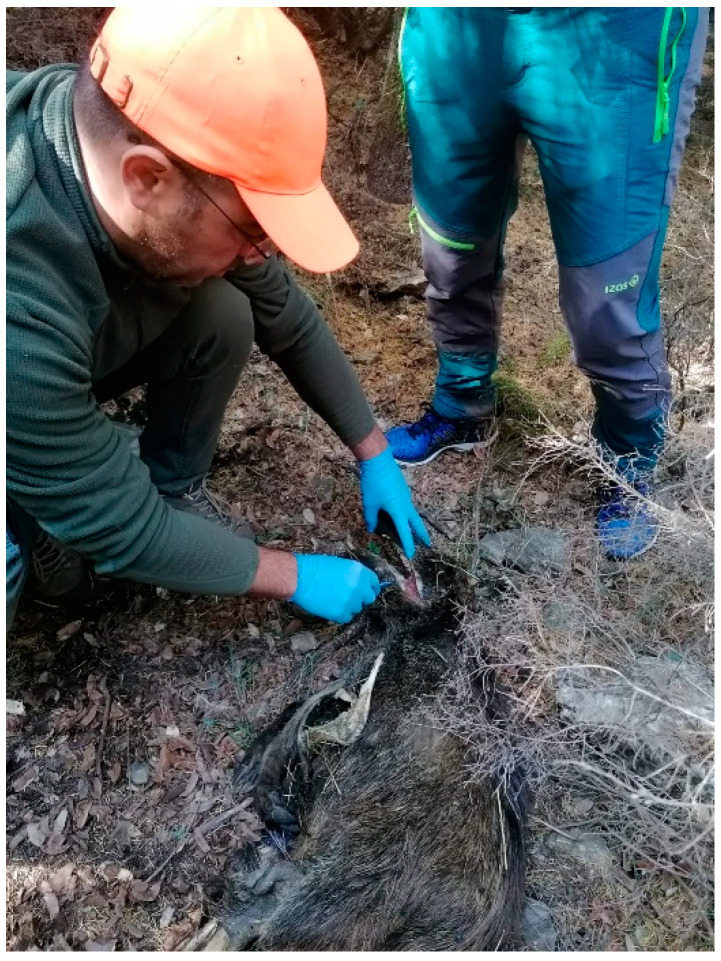
People involved in patrol activities during carcass discovery.

At the location, the team was informed about the area to be covered, possible carcasses already reported, the sub-division of the territory, the presence of private properties, the expected duration of the search, and the final meeting point. People were equipped with high-visibility caps or jackets, transceivers, gloves, and sampling equipment (Figure 5).

When a wild boar carcass was found, the person who found it was required to register the GPS point of the finding, inform the veterinarians of the team by means of phone or transceivers, and upload the data onto the *GAIA observer* app. Veterinarians were responsible for collecting samples from carcasses in compliance with biosecurity measures, storing the samples, and sending them to the IZS-Sardinia laboratories. The samples collected from carcasses were subjected to the same procedure applied to all samples collected from hunted wild boars, undergoing viral DNA search via real-time PCR, as established by the OIE diagnostic manual [47]. 

The conservation status of each carcass (i.e., fresh, decomposing, or mummified) was assessed during sample collection [53]. Furthermore, the veterinarians were responsible for uploading data related to the location (region, province, municipality, latitude, and longitude), sampling date, age, and sex of the carcasses (observed or hypothesized), and the type of sample (i.e., spleen, blood, tonsil, kidney, or lymph node) in the national animal disease database for passive surveillance (SINVSA).

### 2.7. Data Collection and Analyses

A specific database was created that included the data recorded by the *GAIA observer* app and was available for download in Excel.csv format. Each record was checked to evaluate its validity (i.e., the app was switched on during the activities and switched off after, the app was only used when walking, and the app was stopped when the user was in a car). Any incongruency was corrected after consultation with the user.

Correspondence analysis was performed to evaluate the data collected by the *GAIA observer* app and recorded in SINVSA by the veterinarians.

A simple descriptive statistical analysis was carried out to summarize the number of on-field sessions, the number of people with or without dogs, the time spent, the distance traveled, and the number of carcasses found. The name and surname of the operator, municipality, and date were used as keys to match the carcass database (report database) and the route database (real-time route monitoring). 

## 3. Results

The formal actions to provide evidence of ASFV absence by passive surveillance (including all its components reported in Figure 2) started in 2020. Considering the impute parameters used to estimate the number of carcasses, a total of 2, 1, and 3 carcasses were expected during the screening phase in Goceano–Gallura, Nuoro–Baronia, and Gennargentu–Ogliastra HMUs, respectively. During the confirmatory phase, at least 5 carcasses in Goceano–Gallura, 3 in Nuoro–Baronia, and 8 in Gennargentu–Ogliastra HMUs had to be sampled and tested ASFV-negative each year to declare the area free from ASF.

From 2020 to 2023, a total of 183 wild boar carcasses were found and sampled in Sardinia, both inside (74, 40%) and outside (109, 60%) the infected area, as reported in Table 2.

Specifically, when the passive surveillance activities were put in place (2020), a total of 26 wild boar carcasses were found mainly by the Forest Corp patrol activities, of which 7 (27%) were inside the infected area and 19 (73%) were outside. In 2021, 48 carcasses were collected (21, 44% inside and 27, 56% outside the infected area), 67 in 2022 (27, 40% inside and 40, 60% outside the infected area), and 42 in 2023 (19, 4% inside and 23, 55% outside the infected area). Of these 183 overall carcasses, 24 were collected during the active search using the *GAIA observer* app. The other 161 were found by the Forest Corp during their patrol activities or by citizens who voluntarily informed the Forest Corp and were sampled by veterinary services (carcasses type B, Figure 2, bundle B). 

**Figure 6:**
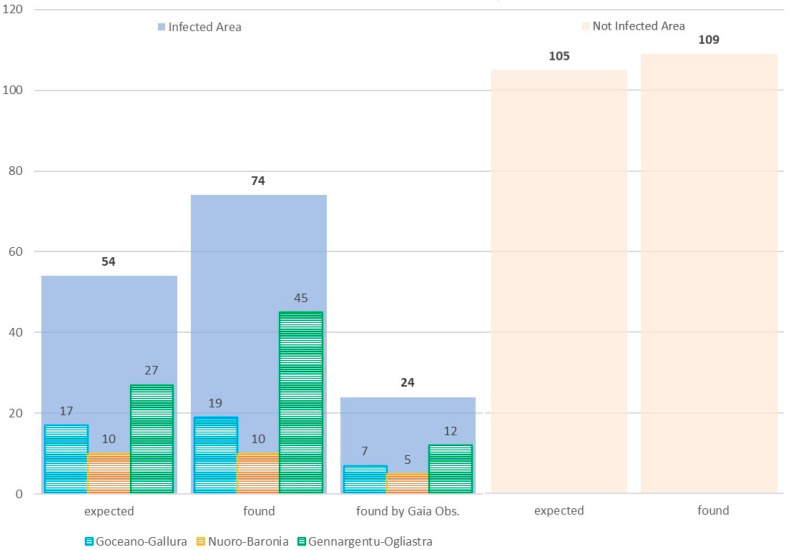
Carcasses expected, found in 2020–2021 and found by *Gaia observer* app between 2021 and 2023 in infected (light blue boxes) and not infected areas (light orange boxes). A detail about the data referring to the three HMUs included in the infected area is proposed.

Figure 6 illustrates the proportion of wild boar carcasses expected, found, and found by the *Gaia observer* app, for each HMU included in the infected area and outside the infected area.

In view of the wild boar carcasses expected during screening and the confirmatory phases, the carcasses found inside and outside the infected area largely provided evidence of virus absence: in the face of 54 expected, a total of 74 carcasses were actively searched and tested as ASFV negative in the three HMUs included in the infected area. A total of 24 (32%) carcasses were found by the *Gaia observer* app. Outside the infected area, passive surveillance started in 2021. To provide evidence of ASFV absence, 35 wild boar carcasses were expected every year, 105 were expected during 2021–2023, and 109 were found and tested as ASFV-negative. 

**Figure 7:**
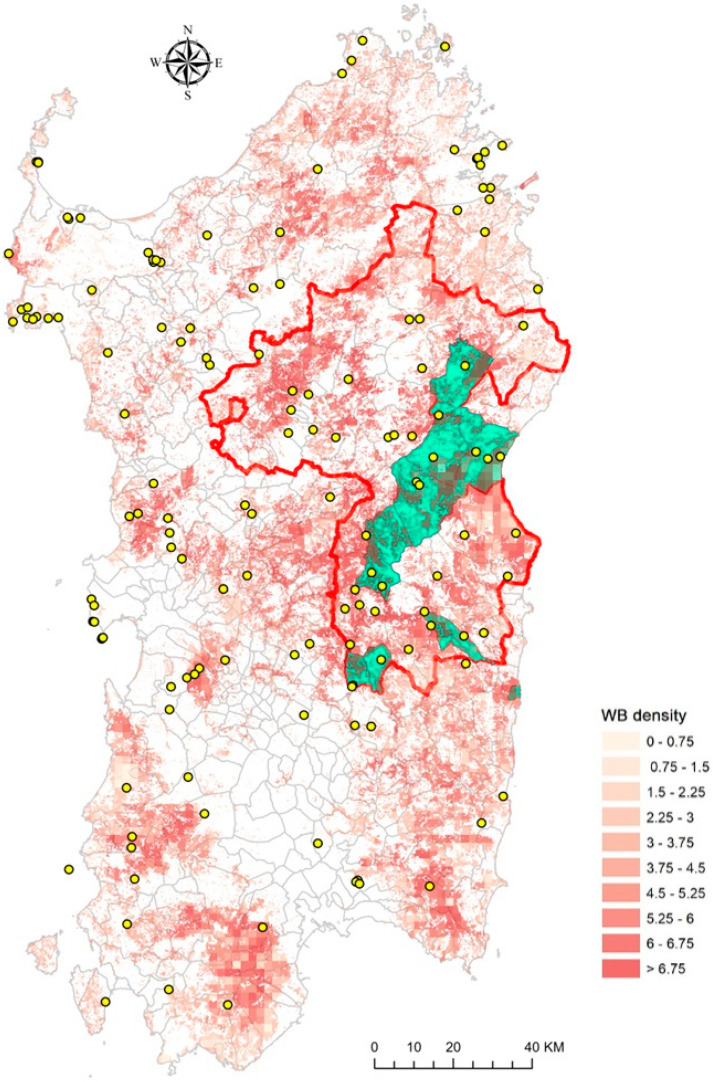
Sardinian map summarizing the geographical distribution of wild boar carcasses and patrol activities. The points indicate the carcasses found during 2021–2023, inside and outside the infected area for 2021 (red line). The green areas indicate the municipalities where the patrol activities were carried out with the use of the *GAIA observer* app. The red gradient indicates the wild boar density.

To obtain the ASFV-free status, the organized patrol activities were mainly focused on the infected area, as represented in Figure 7. 

During the three years of the project, 54 users downloaded the *GAIA observer* app. For the purpose of these analyses, a total of 235 records were downloaded from the *GAIA observer* app for each user during the 6, 11, and 19 on-field activities carried out in 2021, 2022, and 2023, respectively. Some 21% of the records (35 records) presented anomalies: distance coverage equal to zero, duration lower than ten minutes, proportion between distance coverage, and duration higher than 4 km/h (human average speed). Few of these records (9, 5% of the total) were correct after consultation with the user, while the other 26 (15% of the total) were excluded from the analysis. The summarized data on the collected carcasses during the 36 active searches are reported in Table 3. 

The six active searches for carcasses carried out in 2021 were useful for understanding that several non-experts cover an excessively small area and take a long time to cover it: up to a maximum of 22 non-experts were involved in exploring a total surface of about 17 km (average speed in 2021 = 0.56 km/h). No wild boar carcasses were found in 2021 through these activities. 

By 2022, the protocol involved smaller teams (about five to seven experts) with one or two trained dogs. An average speed of 1.3 km/h was reported by the participants. Furthermore, considering that the activities organized in 2021 to find carcasses based on the distribution model yielded no results, the activities were focused on areas where researchers had been alerted by hunters to the possible presence of wild boar carcasses. A total of 12 carcasses were found in 2022 during active searches for 5 carcasses, all of which had previously been discovered by hunters (carcass type A, Figure 2, bundle A).

**Figure 8:**
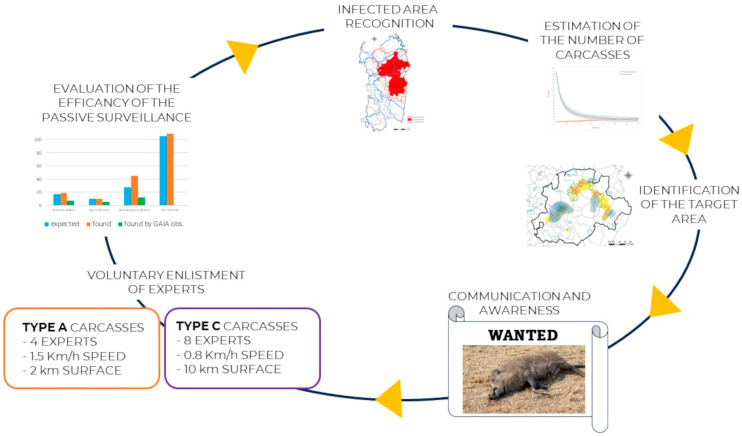
Workflow summarizing the phases of the passive surveillance process.

In 2023, the protocol was further refined: to catch up with the previously reported carcasses, only four expert people were generally involved in the active searches for carcasses to follow up with the previously reported ones. They achieved an average speed of 1.5 km/h, and about 2 km of distance was covered. Even less time was spent when the dog was present, as reported for the activity on 1 August 2023, during which time only 1.2 h were required. If the carcasses were not reported, at least eight expert people would be necessary to cover about 10 km at an average speed of 0.8 km/h. The workflow reported in Figure 8 illustrates the phases of the standardized protocol applied for efficacy in passive surveillance, from the recognition of the infected area to the enlistment of experts based on the carcass types.

**Figure 9:**
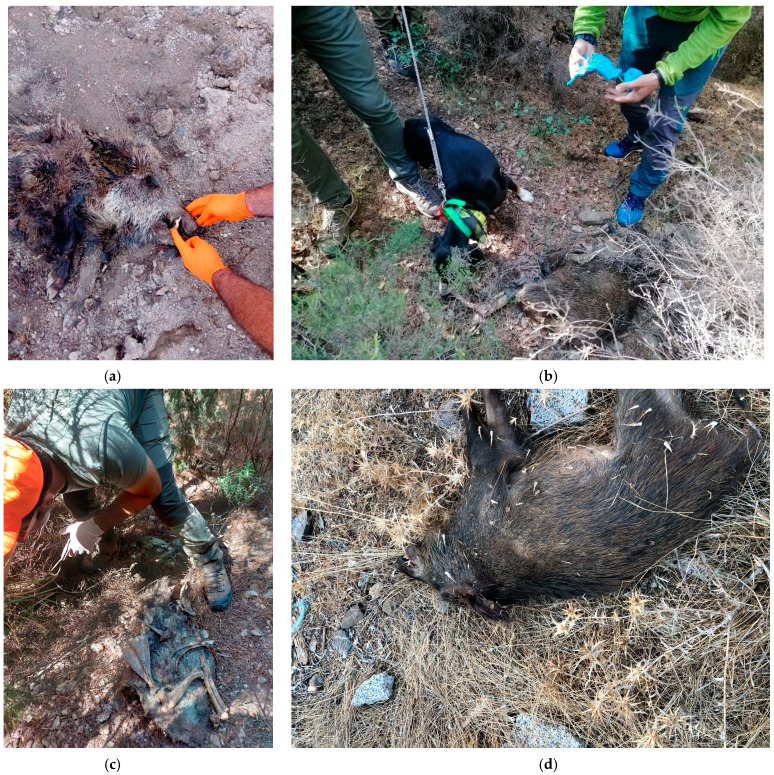
Patrol activity results: (**a**–**c**) wild boar carcasses found in advanced stages of decomposition; (**d**) wild boar fresh carcasses found on 16 March 2023.

A total of twelve carcasses were found in 2023 during 19 organized active searches. Only four carcasses not previously reported were found during this year, which were classified as carcasses of type C (Figure 2, bundle C). Most of the carcasses were found in humid areas (i.e., near waterholes or rivers) in advanced stages of decomposition (i.e., mummified) (Figure 9a–c). Thus, only bone marrow samples were collected. Only seven fresh carcasses (Figure 9d) were found in October 2022 and March 2023, and samples from organs (i.e., spleen, kidney, and lymph nodes) were collected and tested for ASFV.

## 4. Discussion

The international spread of the ASF virus is adversely affecting animal health and welfare, resulting in economic losses of tens of billions of euros per year and potentially affecting livelihoods [54]. The situation is also complicated by the absence of any validated strategy with which to define an area as ASF-free following the detection of the virus in the wild boar population and upon the virus becoming undetectable under the usual surveillance conditions (hunting and opportunistic finding of wild boar carcasses) [31]. 

In the final stage of the ASF eradication process, there is a shadowed time period during which the virus is no longer detectable because of its extremely low prevalence and because the number of carcasses found is also very low due to the possible absence of the virus and related mortalities. In such epidemiological situations, the probability of detecting an ASF-positive wild boar is much higher in animals found dead than in hunted animals [11,17,47].

Using the EFSA exit strategy, it is possible to define a wild boar population as ASF-free despite the sporadic presence of seropositive adult wild boar that could represent the normal evolution of an infection whose agent has been eradicated [17,31]. Based on this strategy, an effective passive surveillance plan must be implemented. Sardinia is the only region affected by ASF to start and complete the exit strategy via active searching for carcasses, obtaining ASF-free status in the wild boar population [38]. The data presented describe the protocol carried out to actively search for, find, and sample the wild boar carcasses needed to provide evidence of ASFV’s absence.

The active search for wild boar carcasses (carcass types A or C) requires the involvement of several people and huge economic resources [42,43,44]. The results of this study confirmed the key role of actively searching for and removing carcasses and eradicating ASF in combination with hunting activities [45] and the importance of implementing, even over the long term, user-friendly apps for efficient data collection. Furthermore, this research demonstrates that the dense vegetation in Mediterranean habitats makes it difficult to search for carcasses and that only expert and motivated people can carry out these searches. Among experts, hunters are the easiest to get involved with. They are the most regular visitors, especially given that often they are also farmers and mushroom gatherers. Moreover, the hunters in Sardinia not only know their profession very well but are censored for the authorization of the hunting season. As such, they are easy to contact and gather [46].

During the active search for carcasses, several problems were faced on account of the rough vegetation, the availability of research, and the typical Sardinian climate. Specifically, the possible presence of carcass type A in a larger or smaller area was reported by hunters or citizens, and an active search for carcasses was organized in this area. Otherwise, those people who searched for the carcasses were not the same as those who notified authorities of them. Thus, when the precise location of the reported carcasses was available, the patrols needed a very short time to find them, supported by georeferenced data via the *GAIA observer* app. 

Finally, even if the presence of the dogs is not always guaranteed, the results confirm that dogs can be applied to search for carcasses. Hunting dogs without special training can be employed with good results, as previously stated [43].

The results of this study highlight that, in order to be successful, passive surveillance requires the employment of several people for a short period. Thus, the target approach must consider the social context and the resources needed/available in terms of the associated costs [20].

In Sardinia, several training courses were established for hunters, giving information on ASF, the way to report wild boar carcasses, the importance of passive surveillance aimed at ASF eradication from wild boar, and their paramount role in collecting and reporting the highest number of carcasses. The awareness process includes transmission via social media (i.e., Facebook), posting leaflets in the locations frequented by hunters (i.e., wild boar handling points), and sending out dedicated mailing lists (Supplementary Materials Figure S1).

Thus, the willingness and motivation of hunters to support passive surveillance is of the utmost importance when using participatory methods [41]. Citizen science and participatory methods can enable wider coverage, but building an efficient disease monitoring system that relies on hunters is the major challenge [37].

## 5. Conclusions

The design of a sufficiently sensitive ASF surveillance system requires a solid understanding of the epidemiology related to the local eco-social context, allowing the generation of a risk-based model and thus making optimal use of the usually limited financial and human resources [7,33]. If stakeholders understand the value of the data collected and the impact of surveillance on animal and human health, this should lead to a better design of surveillance activities, greater compliance with data collection, a greater probability of investment, and achieving the overall goal of surveillance to enable the development of the “one health” approach [34]. 

Community involvement in scientific processes has the potential to combine useful data collection with awareness-raising and education, helping to bridge the knowledge gap between academia and the general public [35], and well-designed citizen science projects can usefully inform research, decision making, and policy formation [36].

Surveillance of wildlife diseases poses considerable logistical challenges compared to that of humans or livestock [37]. In contrast to traditional epidemiology, citizen science, with its passive surveillance model and innovative participatory approach, engages law enforcement, farmers, hunters, trekkers, birdwatchers, and ordinary citizens in scientific research, facilitating the collection of large data sets and increasing public awareness [38]. 

Hunters, a special and often overlooked group of citizen scientists [39], offer unique advantages given their knowledge of the territory, facilitating the collection of long-term data over large areas that otherwise could not be possible to investigate [40]. Thus, researchers and hunters should be considered a standard partnership [44]. Hunters play an important role in wildlife management and, thus, also in the implementation of disease control measures in the wild boar population, especially in the enforcement of ASF control measures and passive disease surveillance [41].

## Figures and Tables

**Table 1:** Parameters used to calculate the number of wild boar carcasses needed by the WBC counter tools for each wild boar hunting management unit (WB-HMU) included in the 2021 infected area.

	Goceano–Gallura	Nuoro–Baronia	Gennargentu–Ogliastra
Wild boar density (wild boar/km^2^)	3.5	3.5	3.5
Surface (km^2^)	1736	1089	2497
Duration of the screening phase (months)	9	8	7
Duration of the confirmatory phase (months)	10	10	11

**Table 2:** Passive surveillance data from 2020 to 2023 for the whole Sardinian region. Data are presented as numbers (percentage), distinguishing between the infected and non-infected areas, with a focus on the three HMUs included totally or partially in the infected area.

Year	Infected Area			Not Infected Area	Total
	Goceano–Gallura	Nuoro–Baronia	Gennargentu–Ogliastra		
2020	2 (8%)	2 (8%)	3 (11%)	19 (73%)	26
2021	6 (12%)	4 (8%)	11 (23%)	27 (56%)	48
2022	9 (13%)	3 (5%)	15 (22%)	40 (60%)	67
2023	2 (5%)	1 (2%)	16 (38%)	23 (55%)	42
Total	19 (10%)	10 (6%)	45 (25%)	109 (60%)	183

**Table 3:** Detailed data on patrol activities carried out in 2021–2023 with the use of the *GAIA observer* app. Data are presented as the number of humans involved, the number of dogs eventually involved, the surface explored by humans and dogs (km), the duration of the session (h), the speed of humans (km for h), the number of wild boar carcasses eventually found, and the carcass type based on 2.3 categories.

Data	Persons Involved	Dogs Involved	Surface Explored by Humans (km)	Surface Explored by Dogs (km)	Total Duration (h)	Average Human Speed (km/h)	Number of Carcasses Detected	Type of Carcass *
13 May 2021	22	0	17.15	0	2.78	0.28	0	-
27 May 2021	15	0	22.75	0	3.59	0.42	0	-
16 June 2021	14	0	9.90	0	3.41	0.21	0	-
29 June 2021	8	0	23.75	0	4.23	0.70	0	-
14 October 2021	4	0	13.03	0	3.25	1.00	0	-
21 October 2021	11	0	22.99	0	2.88	0.73	0	-
17 May 2022	7	2	13.15	26.15	3.31	0.57	4	A
28 May 2022	5	0	11.11	0	3.23	0.69	0	-
7 June 2022	4	0	10.92	0	2.37	1.15	1	A
15 June 2022	4	0	7.14	0	2.45	0.73	2	A
16 June 2022	5	0	9.42	0	2.78	0.68	0	-
5 August 2022	8	0	15.16	0	4.10	0.46	0	-
13 August 2022	11	1	17.99	18.51	3.06	0.53	0	-
15 September 2022	8	0	12.85	0	2.15	0.75	0	-
16 September 2022	8	0	31.57	0	4.38	0.90	0	-
10 October 2022	5	0	5.10	0	0.55	1.86	1	A
28 October 2022	3	0	1.50	0	1.10	0.45	4	A
16 March 2023	4	0	9.47	0	1.59	1.49	2	A
4 May 2023	4	1	5.74	7.41	1.28	1.12	0	-
3 June 2023	7	0	38.01	0	3.18	1.71	0	-
1 August 2023	6	1	21.68	12.12	1.22	2.96	2	A
2 August 2023	2	0	3.15	0	0.55	2.86	0	-
6 August 2023	10	0	25.79	0	2.59	1.00	0	-
9 August 2023	3	0	7.46	0	1.32	1.88	2	A
10 August 2023	5	0	13.68	0	2.23	1.23	0	-
13 August 2023	4	0	9.56	0	1.75	1.36	0	-
16 August 2023	3	0	15.02	0	3.15	1.59	0	-
22 August 2023	5	0	13.12	0	2.48	1. 05	1	C
1 September 2023	18	1	51.90	22.76	3.25	0.89	0	-
18 September 2023	12	1	28.00	14.55	3.02	0.77	0	-
25 September 2023	3	0	2.06	0	0.45	1.52	2	A
29 September 2023	8	0	15.90	0	2.23	0.89	1	C
7 October 2023	1	1	1.43	5.26	1.14	1.25	0	-
10 October 2023	10	0	23.56	0	3.25	0.72	1	C
11 November 2023	8	0	18.01	0	2.59	0.87	1	C
1 December 2023	5	0	6.66	0	1.67	0.79	0	-

* Type A refers to previously reported carcasses, and type C refers to carcasses never reported and found during the patrol activities.

## Data Availability

All the data are reported in the main text.

## Supplementary Materials

The following supporting information can be downloaded at https://www.mdpi.com/article/10.3390/v16010136/s1, Figure S1: Awareness poster on passive surveillance in wild boar against African swine fever, disseminated to Sardinian hunters.
