# Supplementary material for: Passive Surveillance as a Key Tool for African Swine Fever Eradication in Wild Boar: A Protocol to Find Carcasses Tested and Validated in the Mediterranean Island of Sardinia
Source: Viruses. 2024 Jan 18;16(1):136. doi: 10.3390/v16010136 (PMC10820949; doi:10.3390/v16010136)
Supplement: Supplementary file 1 [file viruses-16-00136-s001.zip › Figure S1.pdf]

ASF

African swine fever

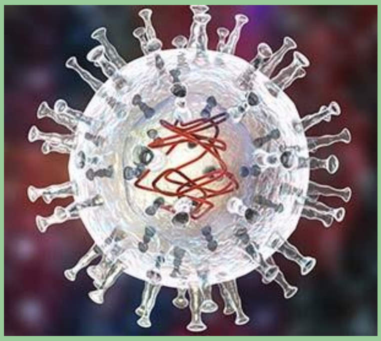

Veterinary Service

Sassari tel. 079.2062813
Nuoro tel. 0784.240010
Oristano tel. 0783.317767
Cagliari tel. 070.6092705

African Swine Fever (ASF) is a highly contagious disease of domestic pigs and wild boars. There is no vaccine to fight it. It is not a danger to human health but is a danger to our agricultural economy leading to severe economic losses

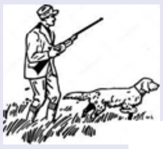

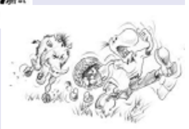

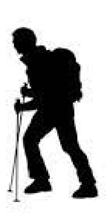

Virus is highly resistant in the environment

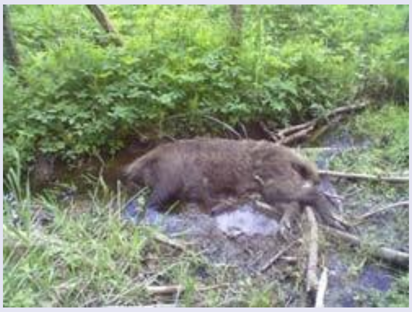

We all play a key role in preventing the spread of African Swine Fever

1515

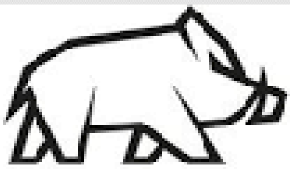

Finding wild boar carcass

What to do?

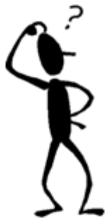

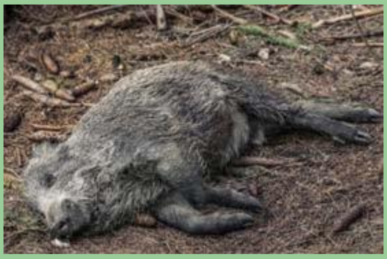

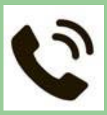

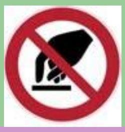

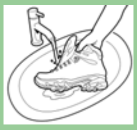

1) Do not touch the carcass

2) Call the ASSL Veterinary Service immediately

3) Store your position on your mobile phone

4) Take a photo

RESPECT GENERAL PRECAUTIONS

Report any suspected cases to the Veterinary Service

Don't touch Don't move the carcass

Disinfect your shoes before getting into the car

CORPO FORESTALE E DI VIGILANZA AMBIENTALE

ATSSardegna Azienda Tutela Salute
